# Insufficient quality of sputum submitted for tuberculosis diagnosis and associated factors, in Klaten district, Indonesia

**DOI:** 10.1186/1471-2466-9-16

**Published:** 2009-05-08

**Authors:** Mateus Sakundarno, Nurjazuli Nurjazuli, Sutopo Patria Jati, Retna Sariningdyah, Sumarsono Purwadi, Bachti Alisjahbana, Marieke J van der Werf

**Affiliations:** 1 Department of Epidemiology, Faculty of Public Health, Diponegoro University, Semarang, Indonesia; 2 Department of Environmental Health, Faculty of Public Health, Diponegoro University, Semarang, Indonesia; 3 Department of Health Policy and Administration, Faculty of Public Health, Diponegoro University, Semarang, Indonesia; 4 District Health Office of Klaten, Central Java, Indonesia; 5 District Health Office of Wonogiri, Central Java, Indonesia; 6 Department of Internal Medicine, Medical Faculty, Padjadjaran University, Hasan Sadikin Hospital, Bandung, Indonesia; 7 TB Operational Research Group, National TB Program, Ministry of Health, Indonesia; 8 KNCV Tuberculosis Foundation, The Hague, the Netherlands; 9 Academic Medical Center, University of Amsterdam, Amsterdam, the Netherlands

## Abstract

**Background:**

Sputum smear microscopy is the standard diagnostic method for detection of smear positive pulmonary tuberculosis (TB). Insufficient quality of sputum might result in missing cases. In this study we aimed at assessing the quality of sputum in a district in Central Java and determining patient and health worker factors associated with submission of three good quality sputum samples.

**Methods:**

In 16 health centers information was collected on the quality of sputum submitted by TB suspects, i.e. volume, color, and viscosity. TB suspects were interviewed to assess their knowledge of TB, motivation to provide sputum and whether they were informed why and how to produce a sputum sample. Health workers were interviewed to assess what information they provided to TB suspects about the reason for sputum examination, methods to produce sputum and characteristics of a good quality sputum sample. All health worker and patient factors were evaluated for association with sputum quality.

**Results:**

Of 387 TB suspects, 294 (76.0%) could be traced and interviewed, and of 272 (70.3%) information about sputum quality was available. Of those 203 (74.6%) submitted three samples, 90 (33.1%) provided at least one good sample, and 37 (13.6%) provided three good quality sputum samples. Of the 272 TB suspects, 168 (61.8%) mentioned that information on the reason for sputum examination was provided, 66 (24.3%) remembered that they were informed about how to produce sputum and 40 (14.7%) recalled being informed about the characteristics of good quality sputum. Paramedics reported to provide often/always information on the importance of sputum examination, and when to produce sputum. Information on how to produce sputum and characteristics of a good sputum sample was less often provided. None of the studied patient characteristics or health worker factors was associated with providing good quality sputum.

**Conclusion:**

A considerable number of TB suspects did not provide three sputum samples and a large number of sputum samples were of insufficient quality. Training of health workers in providing health education to the TB suspect about the reason for sputum examination and how to produce a good quality sputum sample should be a priority of the TB program.

## Background

Indonesia ranks third in number of notified tuberculosis (TB) cases in the world, after China and India [1]. The DOTS program has been implemented since 1995 and covered 98% of the population in 2005 [1]. In 2005, there were an estimated 240,000 smear positive pulmonary TB cases and 93,000 deaths due to tuberculosis. In the same year the national case detection rate (CDR) of new smear positive patients had reached 66% [1].

Sputum smear microscopy is the standard diagnostic method for detection of smear positive pulmonary TB in the DOTS strategy. In settings with limited resources, such as health centers, it is considered a cost effective and appropriate method [2]. Researchers and public health professionals have repeatedly advocated the use of this method [3,4]. In the guidelines of the Indonesian Ministry of Health smear microscopy is the preferred method for the detection of TB [5]. The quality of the microscopy results depends on the quality of the sputum sample and the quality of the microscopic examination [6]. Much work has been done to ensure high quality of microscopic examination by implementation of quality assurance measures. Only few studies have assessed the quality of sputum and factors related to quality. Adequate education of TB suspects by the health care provider has been shown to result in a substantial increase in the quality of the sample, the number of positive samples and the density of acid fast bacilli (AFB) per sputum slide [6-8]. Health service provider factors such as the experience and skill in motivating patients to provide a good sputum sample, and attitude and compliance with sputum collection procedures, may influence the number and quality of sputum samples provided by TB suspects. Also patient related factors such as gender and social economic status may influence the quality of the sputum sample.

In this study, we collected information about characteristics of sputum routinely collected from TB suspects in health centers in Klaten district, Indonesia. We assessed the quality of the sputum samples and we assessed which factors contributed to the quality. We choose to do the study in Klaten district because it is one of the 35 districts in Central Java province with the lowest case detection rate (CDR was 15.3% in 2003) [9].

## Methods

In 2004 there were 34 health centers in Klaten district; 7 laboratory referral health centers; 2 independent laboratory health centers; and 25 satellite health centers. At satellite health centers laboratory technicians collect sputum samples from TB suspects and prepare the slides. The slides are sent to a laboratory referral health center for microscopic examination. Laboratory referral health centers and independent laboratory health centers collect and examine sputum samples. We selected the four laboratory referral health centers and 12 satellite health centers that reported the highest number of TB suspects in the two years before the study (2002 and 2003).

The TB05 form (National TB program form for ordering sputum examination for TB and recording the result) collects information about color and viscosity of the sample and the sputum examination result. To be able to obtain information about the volume of the sample we modified the TB05 form. We also modified the TB06 form (National TB program form that lists the TB suspects identified in the health center) so that it included space to write detailed address information and the laboratory register number to enable linking with the TB05 form. End of September 2004, we organized a one day training session for the doctors, paramedics and laboratory technicians responsible for the TB program to familiarize them with the study, to introduce the modified forms and to teach them how to assess the volume, color and viscosity of a sputum sample. To be able to assess the effect of the introduction of the modified forms on sputum quality we collected the TB05 forms from July, August, and September 2004, the three months before the introduction of the modified forms.

Before the training on how to use the modified forms, we interviewed a doctor, paramedic and laboratory technician in each health care facility about whether and how they motivate suspects to produce sputum, and what information is provided to the patient. According to the National TB manual, the laboratory technician or paramedic is responsible for providing the specimen container to the TB suspect and explaining how to produce a good sputum specimen [5].

From October 1, 2004 the modified forms were introduced and the health centers were visited once a week to collect the TB05 and TB06 forms until end of March 2005. Individuals that were registered as suspect on the TB06 form were eligible for interview. The doctor or paramedic informed the suspect about the study and asked informed consent during the initial registration of the TB suspect. We aimed at interviewing TB suspects as soon as possible after registration. They were traced and interviewed in their home. We used a questionnaire with open and close-ended questions about whether TB suspects knew why they had to produce sputum and how, who provided this information, and their attitude towards sputum collection. If the TB suspect was absent at the first visit the visit was repeated until the suspect was found. When the interviewer did not find the address of the suspect the name and address of the suspect were crosschecked with the head of village. If it was not possible to get the correct address the suspect was not interviewed. The tracing and the interviews were conducted by research assistants under supervision of the researchers. The research assistants were recruited from the school of public health and trained in interviewing TB suspects using the questionnaire.

From the unmodified and modified forms we retrieved information about the number of sputum samples provided by the suspects, the color, the viscosity (sputum or saliva), and the sputum examination result. From the modified forms we also retrieved information about the volume of the sample. This allowed us to assess the quality of sputum collection for all suspects registered after introduction of the modified TB05 form. Quality was considered good if it was complete (i.e. submission of three sputum samples [spot-morning-spot]) and if the quality of the three samples was good, i.e. volume at least 3 ml; color yellowish-green (purulent); and appeared to be sputum (i.e. viscous). We also used a less strict definition of good quality sputum collection, i.e. good if at least one sputum sample of good quality was submitted.

We assessed whether characteristics of the TB suspect such as sex, age, marital status, income, knowledge of TB signs and symptoms, perceived seriousness of TB, motivation to provide sputum, and accessibility of the health center were associated with the quality of the provided sputum. Knowledge was considered 'good' if the TB suspect provided the correct answer to ≥50% of the questions and 'poor' if <50% of the questions was answered correctly. Motivation to provide sputum was considered 'good' if they had a score of more than 13 (range: 0–27) and 'poor' if the score was ≤13.

We also assessed whether information that was provided to the TB suspect by the health worker, as reported by the TB suspect, influenced the quality of the sputum sample.

Data collection tools were pre-tested and adjusted after pre-testing. Data were entered, checked, validated and analyzed in SPSS 11.0 for windows. We used the chi-square test or if appropriate Fisher's exact test for comparison of categorical variables. P-values < 0.05 were considered statistically significant.

The study was provided ethical clearance by the Commission on Health Research Ethics, Faculty of Medicine, Diponegoro University and Dr. Kariadi Hospital Semarang, Indonesia.

## Results

Between June 2004 and April 2005 508 TB suspects were recorded on the TB06 form, 121 (23.8%) before and 387 (76.2%) after the introduction of the modified TB05 and TB06 form. Eighty-nine (17.5%) suspects did not submit any sample, 33 (6.5%) submitted one sample, 23 (4.5%) submitted two samples and 363 (71.5%) submitted 3 samples. It seems that the introduction of modified forms had a small positive effect on the quality of the samples (Table 1). Both the proportion of samples with a yellow-green color, and the proportion that macroscopically appeared to be sputum increased (p < 0.001). The effect on other parameters was not significant. The number of TB suspects who submitted three sputum samples was 79 (65.3%) before and 284 (73.4%) after introduction of modified forms (p = 0.09). Among those who submitted sputum samples, 26 (6.3%) suspects had at least one positive smear, 4 (4.8%) before and 22 (6.5%) after introduction of modified forms (p = 0.56).

**Table 1:** Characteristics of submitted sputum samples and sputum smear results of tuberculosis suspects before and after introduction of modified TB05 forms in the health care facilities in Klaten district, Central Java.

Sputum samples	Before introduction of modified forms (n = 244) n (%)	After introduction of modified forms (n = 924) n (%)	Total (n = 1168) n (%)	P-value
Smear examination result^@^				0.13
Sputum samples smear positive	9 (3.9)	63 (6.9)	72 (6.3)	
Sputum samples smear negative	221 (96.1)	856 (93.1)	1077 (93.7)	

Volume^#^				
Sputum samples with volume ≥3 ml	-*	429 (54.1)		
Sputum samples with volume < 3 ml	-*	364 (45.9)		

Color^&^				<0.001
Sputum samples yellow-green	53 (35.1)	459 (54.0)	512 (51.1)	
Sputum samples with other color	98 (64.9)	391 (46.0)	489 (48.9)	

Viscosity^$^				<0.001
Sputum samples that appear sputum	66 (37.7)	494 (55.4)	560 (52.5)	
Sputum samples that appear saliva	109 (62.3)	397 (44.6)	506 (47.5)	

* Volume of sputum sample was not registered on the original TB05 form therefore quality of the sample can not be assessed before the intervention

^@ ^Of 19 (14 before and 5 after introduction of modified forms) samples the smear microscopy result was not available

^# ^131 sputum samples were not assessed for volume

^&^167 (96 before and 74 after introduction of modified forms) sputum samples were not assessed for color

^$ ^102 (69 before and 33 after introduction of modified forms) sputum samples were not assessed for viscosity

In the interviews that assessed which type of information on sputum collection was provided to the suspect by the health workers, the paramedics reported that they often or always provided health education about the importance of sputum examination, while this was reported by 10 (62.5%) laboratory technicians, and by 13 (81.3%) doctors (Table 2). Paramedics provided more often information about the methods of producing sputum compared to doctors and laboratory workers. All paramedics reported often or always informing the TB suspect to provide two spot samples and one morning sample, 14 (87.8%) often or always provided the TB suspect with information about how to produce a sputum sample, and 12 (75.0%) often or always informed the TB suspect about the criteria of a good sputum sample.

**Table 2:** Type of information provided to tuberculosis (TB) suspects about production of sputum samples for TB diagnosis by health workers in health centers in Central Java.

Type of health education*	Doctors (n = 16) n (%)	Paramedics (n = 16) n (%)	Laboratory technicians (n = 16) n (%)
Provide information about the importance of sputum examination			
Never	2 (12.5)	0 (0.0)	1 (6.3)
Rare	0 (0.0)	0 (0.0)	1 (6.3)
Occasionally	1 (6.3)	0 (0.0)	4 (25.0)
Often	8 (50.0)	6 (37.5)	2 (12.5)
Always	5 (31.3)	10 (62.5)	8 (50.0)

Explain that 3 sputum samples should be produced two on the spot and one in the morning			
Never	2 (12.5)	0 (0.0)	1 (6.3)
Rare	0 (0.0)	0 (0.0)	1 (6.3)
Occasionally	3 (18.8)	0 (0.0)	1 (6.3)
Often	3 (18.8)	2 (12.5)	2 (12.5)
Always	8 (50.0)	14 (87.5)	11 (68.8)

Explain how to produce sputum			
Never	2 (12.5)	0 (0.0)	1 (6.3)
Rare	2 (12.5)	0 (0.0)	1 (6.3)
Occasionally	3 (18.8)	2 (12.5)	5 (31.3)
Often	5 (31.3)	5 (31.3)	5 (31.3)
Always	4 (25.0)	9 (56.3)	4 (25.0)

Explain how a good sputum sample should look			
Never	3 (18.8)	0 (0.0)	1 (6.3)
Rare	2 (12.5)	0 (0.0)	1 (6.3)
Occasionally	5 (31.3)	4 (25.0)	6 (37.5)
Often	4 (25.0)	3 (18.8)	2 (12.5)
Always	2 (12.5)	9 (56.3)	6 (37.5)

* Never: Not one of the examined TB suspects was provided with the information

Rare: Information was provided to <25% of the examined TB suspects

Occasionally: Information was provided to 25–50% of the examined TB suspects

Often: Information was provided to >50% of the examined TB suspects

Always: Information was provided to all TB suspects who were examined.

Of the 387 TB suspects that were registered on the modified TB06 form 294 (76.0%) could be traced and interviewed. The median time between the identification of the TB suspect and the date of interview was 15 days (interquartile range 25 days). Sixty-six TB suspects (17.1%) could not be interviewed because the recorded address was not detailed enough, 12 (3.1%) had moved away, and 15 (3.9%) due to other reasons such as 'died'. Four (1.0%) TB suspects refused to participate in the interview. The sex distribution and age group distribution was comparable in those that could be traced and those that could not be traced (data not shown). The sputum samples of 22 (7.5%) of the 294 interviewed TB suspects could not be evaluated because TB05 did not contain information on volume, color or viscosity; these individuals are not included in the analysis (total n = 272). Approximately half (46.7%) of the interviewed individuals were male (Table 3). The mean age was 48.7 years with standard deviation (SD) of 16.7 years. Two-hundred and two respondents were married (74.3%) and 64 (23.5%) were very poor with an income less than 300,000 rupiah per-month (equals 1 US$ per day).

**Table 3:** TB suspect factors associated with good quality sputum collection, i.e. three samples submitted with volume ≥ 3 ml, color yellow-green, and correct viscosity.

Variable	Individuals with poor quality sputum collection (n = 235)N (%)	Individuals with good quality sputum collection (n = 37) N (%)	Total number of individuals (n = 272)* N (%)	P-value
Sex				0.60
Male	108 (85.0)	19 (15.0)	127 (100)	
Female	127 (87.6)	18 (12.4)	145 (100)	

Age group in years				0.72
15–29	32 (88.9)	4 (11.1)	36 (100)	
30–59	123 (87.2)	18 (12.8)	141 (100)	
≥ 60	80 (84.2)	15 (15.8)	95 (100)	

Marital status				0.60
Married	177 (87.6)	25 (12.4)	202 (100)	
Unmarried	19 (82.6)	4 (17.4)	23 (100)	
Widowed	39 (83.0)	8 (17.0)	47 (100)	

Monthly income in rupiah				0.14
< 300,000	51 (79.7)	13 (20.3)	64 (100)	
300,000–899,999	159 (89.3)	19 (10.7)	178 (100)	
≥ 900,000	25 (83.3)	5 (16.7)	30 (100)	

Knowledge of TB#				0.35
Poor	149 (89.2)	18 (10.8)	167 (100)	
Good	86 (81.9)	19 (18.1)	105 (100)	

Motivation to provide sputum$				0.56
Good	213 (87.3)	31 (12.7)	244 (100)	
Poor	22 (78.6)	6 (21.4)	28 (100)	

Perceived seriousness of TB				0.21
Serious	130 (83.9)	25 (16.1)	155 (100)	
Not serious	105 (89.7)	12 (10.3)	117 (100)	

Perceived walking distance from home to nearest health center				0.13
Close	77 (91.7)	7 (8.3)	84 (100)	
Far	158 (84.0)	30 (16.0)	188 (100)	

Perception of transportation costs to health center				0.36**
Too expensive	8 (72.7)	3 (27.3)	11 (100)	
Reasonable	54 (85.7)	9 (14.3)	63 (100)	
Cheap	171 (87.7)	24 (12.3)	195 (100)	
Don't know	2 (66.7)	1 (3.3)	3 (100)	

Perception of sputum submission				0.15**
Easy	127 (89.4)	15 (10.6)	142 (100)	
Difficult	101 (82.8)	21 (17.2)	122 (100)	
Don't know	7 (87.5)	1 (12.5)	8 (100)	

*the samples of 22 interviewed TB suspects were not evaluated for volume, color and viscosity. These 22 TB suspects are excluded from the analysis

# Knowledge was considered 'good' if the TB suspect provided the correct answer to ≥50% of the questions and 'poor' if <50% of the questions was answered correctly

$ Motivation to provide sputum was considered 'good' if they had a score of more than 13 (range: 0–27) and 'poor' if the score was <13.

** Don't know not included in chi square test

All individuals that were traced and interviewed remembered that they had visited a health center and 168 (61.8%) mentioned that information on the reason for sputum examination was provided (Table 4). This information was most often provided by the paramedic (n = 82, 48.8%), 34 (20.2%) mentioned that the laboratory technician provided the information and 17 (10.1%) the doctor. Thirty-five (20.8%) mentioned that the information was provided by more than one health care worker. Only 66 (24.3%) of the 272 interviewed respondents remembered that they were informed about how to produce a sputum sample. The most frequently suggested method was to drink water (n = 32, 48.5%). Other suggested methods were to take a deep breath (n = 12, 18.2%) or to hold ones breath (n = 3, 4.5%). Only 40 (14.7%) TB suspects recalled being informed about the characteristics of good quality sputum samples. Thirty-five (87.5%) were told that the color of the sputum should be yellow/green. Five (12.5%) recalled being told that the volume of the sample should be at least 3 ml. Information about the quality of a sputum sample was most often provided by the laboratory technician (n = 21, 52.5%). Fourteen (35.0%) TB suspects received the information from paramedics and only 2 (5.0%) received the information from a doctor. Three (7.5%) received the information from more than one health worker. Most (n = 229, 84.2%) TB suspects said not to feel ashamed to produce a sputum sample outside the health center. Also 249 (91.5%) reported not being afraid that others would learn about their disease.

**Table 4:** Association between type of health education received by the TB suspects according to the interview with the TB suspect and the quality of sputum collection, i.e. three samples submitted with volume ≥ 3 ml, color yellow-green, and correct viscosity.

Information provided by health worker	Individuals with poor quality sputum collection (n = 235) N (%)	Individuals with good quality sputum collection (n = 37) N (%)	Total number of individuals (n = 272)* N (%)	P-value
Reason for sputum examination				0.15
Explained	141 (60.0)	27 (73.0)	168 (61.8)	
Not explained	94 (40.0)	10 (27.0)	104 (38.2)	

Method of sputum production				0.22
Explained	54 (23.0)	12 (32.4)	66 (24.3)	
Not explained	181 (77.0)	25 (67.6)	206 (75.7)	

Characteristics of good quality sputum sample				0.80
Explained	34 (14.5)	6 (16.2)	40 (14.7)	
Not explained	201 (85.5)	31 (83.8)	232 (85.3)	

* the samples of 22 interviewed TB suspects were not evaluated for volume, color and viscosity. These 22 TB suspects are excluded from the analysis

Of the 272 TB suspects 37 (13.6%) provided three good quality sputum samples, 90 (33.1%) provided at least one good sample, and 203 (74.6%) provided 3 sputum samples. None of the TB suspect factors about which we have collected information was significantly related to the quality of sputum provision (Table 3). Whether or not information about the reason for sputum examination, the method of sputum production, or the characteristics of a good sputum sample was provided by the health worker according to the TB suspect did not influence the quality of the provided sputum samples (Table 4). Also, if we used the less strict definition of good quality sputum collection we did not find any factor that predicted whether the TB suspect provided good or poor quality sputum (Table 5). Comparing completeness of sample collection (i.e. 3 samples) with all potential explanatory factors showed only low income to be related to incomplete sample collection (Table 6). Those with good quality sputum collection appeared to be diagnosed with smear positive TB (i.e. at least two positive smears) more frequently (strict definition p = 0.02, less strict definition p = 0.12), Table 7 and Table 8.

**Table 5:** TB suspect factors associated with good quality sputum collection using the less strict definition (i.e. at least one sputum sample with volume ≥ 3 ml, color yellow-green, and correct viscosity).

Variable	Individuals with poor quality sputum collection (n = 182) N (%)	Individuals with good quality sputum collection (n = 90) N (%)	Total number of individuals (n = 272)* N (%)	P-value
Sex				0.52
Male	82 (64.6)	45 (35.4)	127 (100)	
Female	100 (69.0)	45 (31.0)	145 (100)	

Age group in years				0.34
15–29	29 (75.0)	9 (25.0)	36 (100)	
30–59	96 (68.1)	45 (31.9)	141 (100)	
≥ 60	59 (62.1)	36 (37.9)	95 (100)	

Marital status				0.48
Married	133 (65.8)	69 (34.2)	202 (100)	
Unmarried	18 (78.3)	5 (21.7)	23 (100)	
Widowed	31 (66.0)	16 (34.0)	47 (100)	

Monthly income in rupiah				0.40
< 300,000	40 (62.5)	24 (37.5)	64 (100)	
300,000–899,999	124 (69.7)	54 (30.3)	178 (100)	
≥ 900,000	18 (60.0)	12 (40.0)	30 (100)	

Knowledge of TB#				0.13
Poor	118 (70.7)	49 (29.3)	167 (100)	
Good	64 (61.0)	41 (39.0)	105 (100)	

Motivation to provide sputum$				0.60
Good	165 (67.6)	79 (32.4)	244 (100)	
Poor	17 (60.7)	11 (39.3)	28 (100)	

Perceived seriousness of TB				0.11
Serious	97 (62.6)	58 (37.4)	155 (100)	
Not serious	85 (72.6)	32 (27.4)	117 (100)	

Perceived walking distance from home to nearest health center				0.52
Close	59 (70.2)	25 (29.8)	84 (100)	
Far	123 (65.4)	65 (34.6)	188 (100)	

Perception of transportation costs to health center				0.50**
Too expensive	6 (54.5)	5 (45.5)	11 (100)	
Reasonable	40 (63.5)	23 (36.5)	63 (100)	
Cheap	134 (68.7)	61 (31.3)	195 (100)	
Don't know	2 (66.7)	1 (33.3)	3 (100)	

Perception of sputum submission				0.66**
Easy	93 (65.5)	49 (34.5)	142 (100)	
Difficult	83 (68.0)	39 (32.0)	122 (100)	
Don't know	6 (75.0)	2 (25.0)	8 (100)	

* the samples of 22 interviewed TB suspects were not evaluated for volume, color and viscosity. These 22 TB suspects are excluded from the analysis

# Knowledge was considered 'good' if the TB suspect provided the correct answer to ≥50% of the questions and 'poor' if <50% of the questions was answered correctly

$ Motivation to provide sputum was considered 'good' if they had a score of more than 13 (range: 0–27) and 'poor' if the score was <13.

** Don't know not included in chisquare test

**Table 6:** TB suspect factors associated with completeness of sputum sample collection (i.e. 3 samples).

Variable	Individuals with incomplete sputum sample collection (<3 samples) (n = 69) N (%)	Individuals with complete sputum sample collection (3 samples) (n = 203) N (%)	Total number of individuals (n = 272)* N (%)	P-value
Sex				0.95
Male	32 (25.2)	95 (74.8)	127 (100)	
Female	37 (25.5)	108 (74.5)	145 (100)	

Age group in years				0.63
15–29	9 (25.0)	27 (75.0)	36 (100)	
30–59	39 (27.7)	102 (72.3)	141 (100)	
≥ 60	21 (22.1)	74 (77.9)	95 (100)	

Marital status				0.78
Married	53 (26.2)	149 (73.8)	202 (100)	
Unmarried	17 (73.9)	6 (26.1)	23 (100)	
Widowed	10 (21.3)	37 (78.7)	47 (100)	

Monthly income in rupiah				0.04
< 300,000	22 (34.4)	42 (65.6)	64 (100)	
300,000–899,999	44 (24.7)	134 (75.3)	178 (100)	
≥ 900,000	3 (10.0)	27 (90.0)	30 (100)	

Knowledge of TB#				0.24
Poor	47(28.1)	120 (71.9)	167 (100)	
Good	22 (21.0)	83 (79.0)	105 (100)	

Motivation to provide sputum$				0.86
Good	61 (25.0)	183 (75.0)	244 (100)	
Poor	8 (28.6)	20 (71.4)	28 (100)	

Perceived seriousness of TB				0.11
Serious	40 (25.8)	115 (74.2)	155 (100)	
Not serious	29 (24.8)	88 (75.2)	117 (100)	

Perceived walking distance from home to nearest health center				0.25
Close	17 (20.2)	67 (79.8)	84 (100)	
Far	52 (27.7)	136 (72.3)	188 (100)	

Perception of transportation costs to health center				0.68**
Too expensive	4 (36.4)	7 (63.6)	11 (100)	
Reasonable	16 (25.4)	47 (74.6)	63 (100)	
Cheap	48 (24.6)	147 (75.4)	195 (100)	
Don't know	1 (33.3)	2 (66.7)	3 (100)	

Perception of sputum submission				0.12**
Easy	29 (20.4)	113 (79.6)	142 (100)	
Difficult	35 (28.7)	87 (71.3)	122 (100)	
Don't know	5 (62.5)	3 (37.5)	8 (100)	

* the samples of 22 interviewed TB suspects were not evaluated for volume, color and viscosity. These 22 TB suspects are excluded from the analysis

# Knowledge was considered 'good' if the TB suspect provided the correct answer to ≥50% of the questions and 'poor' if <50% of the questions was answered correctly

$ Motivation to provide sputum was considered 'good' if they had a score of more than 13 (range: 0–27) and 'poor' if the score was <13.

** Don't know not included in chisquare test

**Table 7:** The smear microscopy result according to the quality of the specimen collection using the strict definition (i.e. three samples submitted with volume ≥ 3 ml, color yellow-green, and correct viscosity).

Smear microscopy results*	Individuals with poor quality sputum sample collection n (%)	Individuals with good quality sputum sample collection n (%)	Total number of individuals n (%)
No sputum sample submitted	30 (12.8)	0 (0.0)	30 (11.0)

Negative (for all samples submitted)	195 (83.0)	31 (83.8)	226 (83.1)

Positive 2×	2 (0.8)	1 (2.7)	3 (1.1)

Positive 3×	8 (3.4)	5 (13.5)	13 (4.8)

Total	235 (86.4)	37 (13.6)	272 (100)

* the samples of 22 interviewed TB suspects were not evaluated for volume, color and viscosity. These 22 TB suspects are excluded from the analysis

**Table 8:** The smear microscopy result according to the quality of the specimen collection using the less strict definition (i.e. at least one sputum sample with volume ≥ 3 ml, color yellow-green, and correct viscosity).

Smear microscopy results*	Individuals with poor quality sputum sample collection n (%)	Individuals with good quality sputum sample collection n(%)	Total number of individuals n (%)
No sputum sample submitted	30 (16.5)	0 (0.0)	30 (11.0)

Negative (for all samples submitted)	145 (79.7)	81 (90.8)	226 (83.1)

Positive 2×	2(1.1)	1 (1.1)	3 (1.1)

Positive 3×	5 (2.7)	8 (8.9)	13 (4.8)

Total	182 (66.9)	90 (31.4)	272 (100)

* the samples of 22 interviewed TB suspects were not evaluated for volume, color and viscosity. These 22 TB suspects are excluded from the analysis

## Discussion

Examination of sputum samples by microscopy is the main method used for diagnosis of tuberculosis in the public health care system of Indonesia. To achieve maximum sensitivity of this diagnostic test it is essential to have a good quality sputum sample, i.e. the sample should contain mucoid or mucopurulent material and the volume should be at least 3 ml [10]. Furthermore, 3 samples should be examined before smear positive tuberculosis can be excluded. In Klaten district only few TB suspects provided 3 samples of good color, viscosity, and volume. We assessed whether this was due to poor health education. Although a high percentage of the TB suspects did not recall being informed about the reason for sputum examination, the method of sputum production and the characteristics of a good sputum sample we did not find this to be related to having submitted three good quality sputum samples or not. It is possible that those who produced poor quality sputum samples did not have lung pathology and were therefore not able to cough up good quality sputum. According to the guidelines of the National Tuberculosis Program of Indonesia a TB suspect is a person with cough for more than three weeks [5]. The person may have other symptoms as well. After our study the definition for a TB suspect has been revised into having productive cough for two weeks or more [11]. If suspect identification is performed properly, a high percentage of suspects should be able to cough up sputum after adequate health education. Also, an adequate TB suspect definition should result in a reasonable percentage of smear positives. According to the National TB manual on average 10% of the examined smears should be positive. [5]. The percentage positive smears in our study was lower. This might be explained by an inappropriate suspect definition. However, it is more likely due to the low quality of sputum samples. The prevalence of HIV is low in our study area (<1%), and does not explain the low percentage of positive smears [12].

We assessed other explanations for the poor quality of sputum such as stigma or transportation difficulties to the health care facility. Stigma does not seem to be an important explanation for the low quality of sputum since most TB suspect respondents did not indicate that they were afraid that their disease would be known by others and they did not find it problematic to cough up sputum outside of the health care facility. Also perceived transportation difficulties or high costs were not related to the quality of sputum collection.

We asked health workers whether they provided information about why and how to produce sputum and about the characteristics of a good sputum sample. We also asked TB suspects whether the information mentioned above was provided by a health worker. The majority of the health workers said that they provided the information. Also, the majority of the TB suspects remembered being informed by the health worker about the reason for the sputum examination. However, only 24% of the interviewed TB suspects remembered being told how to produce a sputum sample, and only 15% remembered that the health worker informed them about the characteristics of a good sputum sample. This discrepancy might be due to recall bias. For most TB suspects the time between identification as TB suspect and the interview was not very long. All TB suspects were interviewed within 6 months after identification as a TB suspect, and for most the time between being identified as a TB suspect and the interview was much shorter than 6 months. It is therefore also possible that although the health workers have provided the information to the TB suspects the information was not provided in a way that was well understood by the TB suspects and remembered. Therefore, training of health workers in providing information about sputum production using methods suitable for the target population is needed. Since paramedics are widely available in the primary care setting in Indonesia and paramedics have frequent contact with patients the training can start with this group.

A study in Nicaragua, in which TB suspects were interviewed one to two months after being identified as TB suspect, showed that most TB suspects remembered that they were informed about why and how to produce a sputum sample [13]. Even though, 46% of the samples were salivary and 40% less than 5 ml. Thus providing information is not sufficient. Therefore, we recommend that health workers check the volume and appearance of the sample when it is submitted. If needed, they can provide additional information and collect a new sample.

Two studies that tested simple interventions for improving the quality of sputum using a randomized trial design showed an increase in the quality of sputum and the yield of positive smears [6,8]. In a study from Indonesia, the intervention especially increased the number of TB suspects that provided a sample of > 5 ml volume [6]. It has been shown that samples of adequate volume are more often positive by smear microscopy [4]. A trial in Pakistan was especially successful in increasing the percentage of women with a positive smear using a two minute instruction by a health worker [8].

The main limitation in our study is that we had to introduce modified forms to be able to measure the quality of the sputum samples and to obtain the address of the TB suspect. The introduction of the modified forms might have influenced the health workers so that they were more aware of the importance of the quality of the sputum sample and provided more information to TB suspects. We did observe a small increase in the percentage of samples with good color and viscosity. So the results of our study might be too positive.

## Conclusion

This study shows that a large number of sputum samples submitted for microscopic examination are of insufficient quality. This may have a significant impact on the number of TB cases diagnosed since the chance of finding bacilli is lower in sputum samples of insufficient quality. Given that most TB suspects do not recall being informed on how to produce sputum we feel that training of paramedics and laboratory technicians, in providing health education to the TB suspect about the reason for sputum examination and how to produce a good quality sputum sample should be a priority of the TB program. Furthermore, continuous supervision and monitoring of the performance of the health workers is crucial to improve and sustain sputum collection.

## Abbreviations

CDR: Case Detection Rate; DOTS: Directly Observed Treatment Short course; ml: Milliliters; SD: Standard Deviation; TB: Tuberculosis; TB05: National Tuberculosis program form for ordering and recording the result of sputum examination; TB06: National tuberculosis program form that list the tuberculosis suspects identified in the health center

## Competing interests

The authors declare that they have no competing interests.

## Authors' contributions

MS participated in design of the study, development of data collection tools and drafting the manuscript. He was responsible for performing data management and for the statistical analysis and interpretation of the data. NN participated in design of the study and development of data collection tools. NN coordinated and data collection. SPJ participated in design of the study and development of data collection tools and assisted in the training of the health workers, coordination of data collection, and data management. SJP participated in the development of data collection tools and coordinated the research assistant training. RS participated in design of the study, development of data collection tools and organized the work in the health centers and coordinated pre-testing of the data collection instruments. SP participated in design of the study and development of data collection tools and assisted in pre-testing of data collection instruments. BA participated in design of the study, and development of data collection tools and monitored data collection and data management and analysis. MJW participated in design of the study, development of data collection tools, monitoring of data collection, data analysis and interpretation and drafting and revising the manuscript. All authors read and approved the final manuscript.

## Pre-publication history

The pre-publication history for this paper can be accessed here:



## References

[B1] WHO (2007). Global Tuberculosis Control: Surveillance, Planning, Financing WHO report 2007.

[B2] Harries AD, Kamenya A, Subramanyam VR, Maher D, Squire SB, Wirima JJ, Nyangulu DS, Nunn P (1997). Screening pulmonary tuberculosis suspects in Malawi: testing different strategies. Trans R Soc Trop Med Hyg.

[B3] Crampin AC, Floyd S, Mwaungulu F, Black G, Ndhlovu R, Mwaiyeghele E, Glynn JR, Warndorff DK, Fine PE (2001). Comparison of two versus three smears in identifying culture-positive tuberculosis patients in a rural African setting with high HIV prevalence. Int J Tuberc Lung Dis.

[B4] Warren JRBM, De Almeida KN, Trakas K, Peterson LR (2000). A minimum 5.0 ml of sputum improves the sensitivity of acid-fast smear for Mycobacterium tuberculosis. Am J Respir Crit Care Med.

[B5] (2003). Pedoman Nasional Penanggulangan Tuberkulosis.

[B6] Alisjahbana B, vanCrevel R, Danusantoso H, Gartinah T, Soemantri ES, Nelwan RHH, Meer JWMvd (2005). Better patient instruction for sputum sampling can improve microscopic tuberculosis diagnosis. Int J Tuberc Lung Dis.

[B7] Hirooka T, Higuchi T, Tanaka N, Ogura T (2004). The value of proper sputum collection instruction in detection of acid-fast bacillus. Kekkaku.

[B8] Khan MS, Dar O, Sismanidis C, Shah K, Godfrey-Faussett P (2007). Improvement of tuberculosis case detection and reduction of discrepancies between men and women by simple sputum-submission instruction: a pragmatic randomised controlled trial. Lancet.

[B9] (2004). Central Java tuberculosis control program feed back report 2003.

[B10] Narvaiz_De_Kantor I, Kim SJ, Frieden T, Laszlo A, Luelmo F, Norval P-Y, Rieder H, Valenzuela P, Weyer K (1998). Laboratory Services in Tuberculosis Control Part II: Microscopy.

[B11] (2007). Pedoman Nasional Penanggulangan Tuberkulosis.

[B12] Herawati L (2005). Situasi HIV-AIDS di Jawa Tengah.

[B13] Macq J, Solis A, Velazquez H, Dujardin B (2005). Informing the TB suspect for sputum sample collection and communicating laboratory results in Nicaragua: a Neglected process in tuberculosis case finding. Salud Publica Mex.

